# Smooth Critical Dimension Compensation Across Photomask Transmittance Discontinuities Enabled by Selective and Direct Laser Patterning Inside Mask

**DOI:** 10.3390/mi17010095

**Published:** 2026-01-11

**Authors:** Dabin Park, Geumsu Yeom, Sungho Jeong, Junsu Park

**Affiliations:** 1 School of Mechanical Engineering & Advanced Machinery Technology Research Institute, Kunsan National University, Gunsan-si 54150, Republic of Korea; qkrekqls020206@kunsan.ac.kr (D.P.); gsyeom@kunsan.ac.kr (G.Y.); 2 Department of Mechanical and Robotics Engineering, Gwangju Institute of Science and Technology, Gwangju 61005, Republic of Korea

**Keywords:** photolithography overexposure, selective laser patterning, critical dimension non-uniformity, photomask transmittance

## Abstract

A selective laser patterning technique applied inside photomasks as a practical method to mitigate critical-dimension non-uniformity caused by overexposure in large-area lithography systems with segmented illumination was investigated. The geometric characteristics of laser-induced voids were analyzed depending on various laser patterning conditions, and the resulting critical-dimension behavior was evaluated across regions with various transmittance levels, including sharply discontinuous transmittance boundaries. The results show that the void size and morphology can be tuned by adjusting the laser pulse energy, although excessive pulse energy leads to mask fracture, from which we derived appropriate processing windows. Furthermore, photomask transmittance was controllable over a wide range (20–92%) by varying laser parameters, void density, pattern arrangement, and the number of patterned layers. This enabled critical-dimension compensation with nanometer- to tens-of-nanometer-level precision. To examine critical-dimension behavior under abrupt transmittance transitions analogous to overexposure zones, 80% and 50% transmittance regions were placed adjacently. Despite the 30% transmittance difference, critical-dimension variation remained smooth, confirming that sharp transmittance changes do not induce abrupt critical-dimension shifts. Overall, our findings experimentally demonstrate that selective and direct laser patterning inside photomasks is a practical and effective critical-dimension compensation approach for large-area lithography employing segmented illumination systems.

## 1. Introduction

Conventional photolithography methods have been employed to fabricate transistors and integrated circuit (IC) chips—key components still prevalent in current microelectronic technologies [1]. Central to planar lithography is the formation of two-dimensional patterns with steep vertical sidewalls. This approach enables the transfer of intricate designs onto substrates, most commonly silicon wafers, to construct the complex circuits essential for device functionality [2]. With ongoing technological advancements, the demand for smaller, faster, and more efficient electronic devices continues to escalate. Photolithography plays a vital role in this trend by enabling the miniaturization of chip features, which in turn allows for a higher density of transistors and other elements within limited space [3,4,5].

Especially in large-area photolithography for display industries, exposure systems are typically equipped with multiple modular light sources to accommodate overexposure regions. While this configuration enables scalability for industrial applications, it inevitably introduces a critical drawback: non-uniform light intensity at the regions where illumination modules overlap. In such zones, excessive or insufficient superposition of light degrades the stability of linewidth control, undermines the uniformity of the critical dimension (CD), and reduces the available process depth of focus [6,7,8].

Over the years, various homogenization techniques have been proposed to mitigate this issue. Among them, the most straightforward and widely adopted industrial solution has been the use of transmittance-control films attached to the photomask surface [9,10]. By measuring the uneven intensity distribution first, a semi-transparent film designed to attenuate light selectively is laminated onto the mask, thereby evening out illumination. This method remains attractive due to its simplicity and relatively low cost. However, despite its practicality, this approach suffers from intrinsic limitations. Films inevitably undergo yellowing or transmittance drift under ultraviolet (UV) irradiation and thermal stress, leading to fading correction performance over time. More critically, such films cannot be repaired when light source conditions change or new non-uniformities appear, local adjustments are impossible, and the entire film must be remanufactured or replaced [9].

To overcome these drawbacks, more advanced strategies aim to engineer the transmittance of the photomask. This can be achieved by generating microscopic modifications, voids, or scattering centers either within the substrate or at its surface [9,11,12,13,14]. In effect, the mask becomes a built-in graded filter, capable of delivering customized homogenization without the films. Among the various approaches, laser-based transmittance control is particularly promising [15,16,17,18]. Laser micropatterning offers some advantages. First, it combines speed with high precision, enabling localized control across large substrates within short processing times. Second, because the induced patterns are embedded in the mask, their optical effect is stable and semi-permanent—free from issues such as film detachment or yellowing. Third, laser-based methods uniquely allow for re-repair. For example, an additional laser processing can correct emerging non-uniformities caused by source aging or contamination, eliminating the need to discard or replace the mask. Taken together, these attributes establish laser patterning as a fundamentally robust alternative to traditional film-based methods, with significant implications for high-resolution manufacturing. In display panel production, for example, where micrometer-scale pattern fidelity is indispensable, such techniques hold substantial promise for both yield enhancement and equipment efficiency.

Despite the long-standing use of laser-patterned photomasks for CD control in large-area, segmented exposure systems, studies on how laser processing conditions shape internal mask patterns, how these patterns influence transmittance, and how they affect actual lithography results remain limited. Research on CD behavior in regions where transmittance is sharply reduced to mitigate local overexposure is particularly scarce.

In this work, we evaluate selective and direct laser patterning as a method to correct CD non-uniformity caused by light-source overlap. We analyze the laser-induced scattering patterns inside photomasks and experimentally measure transmittance changes with respect to pattern pitch, density, and layering. To verify practicality, we perform real exposures using masks containing these patterns and assess CD control and exposure characteristics in regions with multiple pattern types. Most importantly, we experimentally demonstrate CD behavior within sharply reduced-transmittance zones corresponding to overexposed areas.

## 2. Materials and Methods

### 2.1. Sample Preparation

The photomasks used for analyzing laser-induced scattering patterns were fabricated from high-purity quartz (Corning, 99.9999%, Corning, NY, USA) and had dimensions of 80 mm × 15 mm. For accurate characterization of the scattering structures, the samples used for pattern-morphology analysis were polished on all sides to ensure full optical transparency.

The photomasks used for transmittance analysis were made of the same quartz material and had dimensions of 25 mm × 25 mm. To investigate photolithography behavior across a wide range of transmittance levels, a relatively large photomask (126.6 mm × 126.6 mm, 5 inch) was also employed. All quartz samples described above had a thickness of 2.3 mm.

### 2.2. Selective Laser Patterning

The laser used to generate scattering patterns inside the photomask was a Yb:KGW pulsed laser (CB3, LIGHT CONVERSION, Vilnius, Lithuania) with a wavelength of 1030 nm, a pulse width of 250 fs, and a repetition rate of 1 MHz. To analyze the morphology and size of the scattering patterns as a function of laser pulse energy, the pulse energy was varied from 10 to 40 μJ in increments of 10 μJ. An NIR ×50 objective lens (Mitutoyo, Kawasaki, Japan) was employed to focus the laser beam. The transmittance of the unpatterned photomask was 92% at 365 nm, corresponding to the i-line wavelength of the exposure system.

A schematic of the experimental conditions used to reduce the photomask transmittance from 92% down to 20% is provided in Table 1. The spacing of the applied scattering patterns ranged from 2.5 to 10 μm. To control the spacing, the laser repetition rate was fixed at 10 kHz, and the stage scanning speed was adjusted accordingly. For a wider transmittance-control range, the number of scattering-pattern layers increased to either one or two by adjusting the Z-position of the stage to the laser focusing direction. The first and second scattering-pattern layers were positioned at depths of 1.0 mm and 1.2 mm from the quartz surface, respectively. To investigate how the arrangement of scattering-pattern layers affects transmittance, the voids in the first and second layers were arranged either in a parallel or in a staggered alignment. The range of photomask transmittance applied to investigate its impact on the photolithography process was 50–92%, and the corresponding laser patterning conditions used for transmittance control are marked in the section entitled ‘Application of laser patterning conditions for lithography.’

### 2.3. Optical Characterizations

The morphology and size of the scattering patterns formed inside the quartz by laser irradiation were analyzed using an optical microscope (Olympus, STM7-MF, Tokyo, Japan). Changes in quartz transmittance under various laser-processing conditions were measured with a UV–VIS–NIR spectrophotometer (Varian Inc., Cary 5000, Palo Alto, CA, USA). For transmittance evaluation, the patterned scattering area inside the quartz was 20 mm × 20 mm. Among the exposure wavelengths used in photolithography—i-line (365 nm), h-line (405 nm), and g-line (436 nm)—the i-line wavelength most strongly affects resolution, linewidth control, and CD uniformity. Therefore, 365 nm was selected as the reference wavelength for analyzing transmittance changes induced by laser direct patterning.

### 2.4. Photolithography Process

To experimentally evaluate the CD control achieved through transmittances, the laser-processed quartz were fabricated into photomasks through the following sequential processes. The photomask fabrication was carried out using a PR track system (MSX2000, SVS).

(1) Positive photoresist (PR, YPP-1700, YoungChang Chemical Co., Sungju, Korea) coating with thickness of 1.5 μm with 1500 rpm for 30 s
(2) Soft bake at 90 °C for 60 s
(3) Mask alignment and exposure to ultra-violet light source with 50 mJ for 7.3 s
(4) Develop PR with tetramethylammonium hydroxide (TMAH, Sigma-Aldrich, MI, USA) of 2.38% for 60 s
(5) Hard bake at 90 °C for 60 s
(6) Based on the developed PR pattern as a mask, etching the exposed chromium absorber layer
(7) PR strip and cleaning

**Figure 1:**
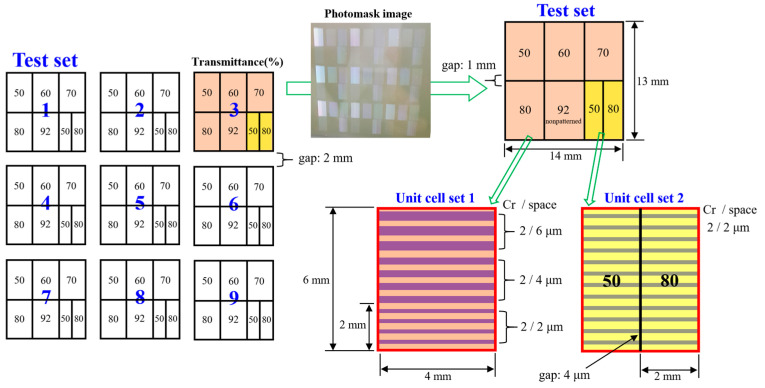
Photomask design schematic to evaluate critical dimension (CD) stability and reproducibility.

The photomask design used to evaluate patterning limits and CD stability with regard to photomask transmittances is shown in Figure 1. The transmittance range applied for CD-compensation testing was 50–92%, increased in 10% increments (test set). Patterns were designed with a target CD of 2 μm, and to analyze diffraction and interference induced by adjacent illumination, the same CD of 2 μm line varied to the space of 2, 4, and 6 μm (unit cell set 1).

To further examine diffraction and interference behavior at sharp transmittance transitions, regions with 50% and 80% transmittance were placed adjacent to each other while maintaining a 2 μm CD and a uniform 2 μm space (unit cell set 2). The distance between layers with different transmittance values was set to 4 μm. For experimental reproducibility and to evaluate CD-compensation variations depending on mask position, a total of 9 test sets were placed at different locations on the photomask, as shown in Figure 1.

After photomask fabrication, exposure was performed using a 365 nm wavelength and an intensity of 20 mW/cm^2^. Following exposure, CD-compensation depending on transmittances was evaluated using a scanning electron microscope (SEM). For each test set, the mean CD value and relative standard deviation (RSD) were calculated from 30 CD measurements obtained across the transmittance and space conditions.

## 3. Results and Discussion

### 3.1. Laser-Induced Void Pattern Characteristics

**Figure 2:**
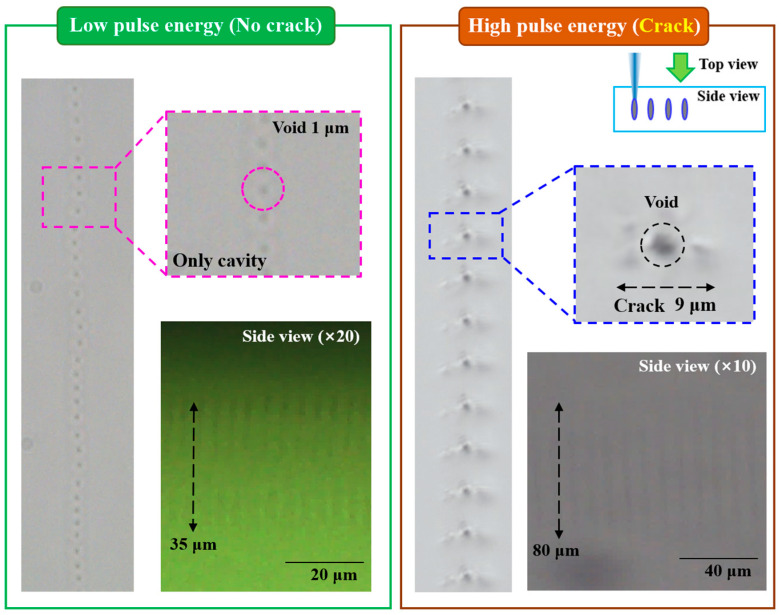
Optical microscope images of void patterns formed inside the photomask by femtosecond laser with regard to pulse energies.

Optical microscope images of the void patterns formed inside the photomask by femtosecond-laser irradiation, along with the corresponding geometric dimensions and transmittance values, are presented in Figure 2 and Table 1, respectively. A laser single-pulse generates a circular void (approximately 1 μm in diameter) at the laser focal point when viewed from the top. A side-view observation reveals an elongated, needle-shaped void (approximately 35 μm in length) extending along the laser propagation direction. Because transparent materials possess a wide bandgap, they do not absorb single-photon infrared energy, allowing the laser beam to pass through the material without interaction. However, when the beam is tightly focused with an extremely short pulse duration, and reaches a high laser intensity (>10^12^ W/cm^2^), multiphoton absorption elevates the local energy beyond the bandgap threshold. This induces electron–hole pair generation and initiates localized deformation, melting, or material breakdown within the transparent medium [19]. Since laser intensity is inversely proportional to pulse width, ultrashort-pulse lasers such as femtosecond sources can readily induce internal modification even at infrared wavelengths.

Analysis of void morphology as a function of pulse energy shows that the void diameter increases (up to ~2 μm) and the void length extends significantly (up to ~80 μm) as pulse energy increases. However, beginning at 30 μJ, an additional region of material modification appears around the void. Because laser intensity is directly proportional to pulse energy, this peripheral modification is attributed to microcrack formation in the transparent material under excessive energy input. Although threshold values vary among researchers due to differences in laser and optical system configurations, similar crack formation and damage phenomena have been reported [20,21]. Transparent materials such as photomasks are inherently brittle; thus, microcracks generated at high pulse energies can significantly reduce their mechanical strength. Because the photomask used in this experiment has a relatively small thickness of 2.3 mm, it is susceptible to fracture during sample preparation or experiment even under weak external forces, such as handing or bending or twisting. Furthermore, excessively long voids limit the number of scattering-pattern layers that can be stacked inside the photomask, restricting the attainable transmittance-control range. Therefore, to safely evaluate transmittances and its lithographic impact under various laser patterning conditions, all laser patterning experiments in this study were performed using pulse energies in the 10–20 μJ range (1270–2540 J/cm^2^), and 1 or 2 patterning layers were applied.

### 3.2. Photomask Transmittance Control

#### 3.2.1. Laser Patterning Density

**Figure 3:**
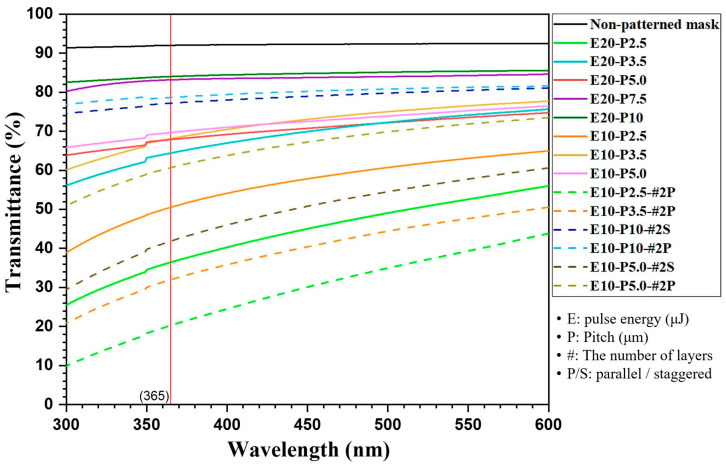
Photomask transmittance curve depending on the wavelength at different laser processing conditions.

**Figure 4:**
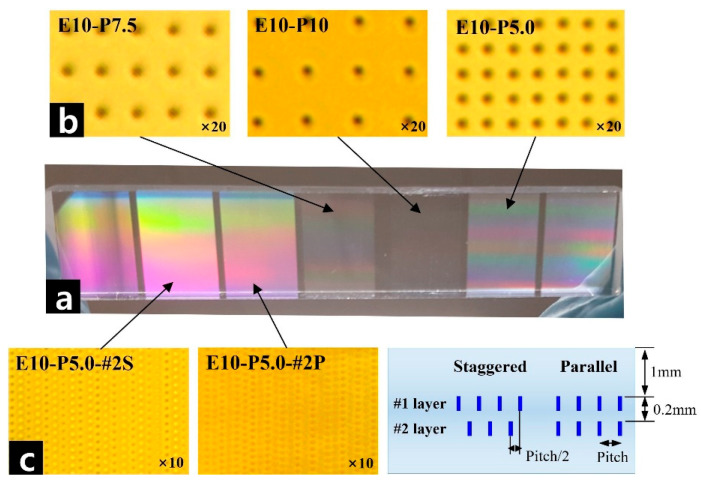
Images of void-pattern distribution and transmittance variation of the transparent material for different pitches and layer arrangements.

Because the spectrophotometer used for transmittance measurements switches its light source between a deuterium lamp and a tungsten–halogen lamp near 350 nm, we note that slight noise appears in the measured transmittance around this wavelength, as shown in Figure 3. The changes in photomask transmittance under various selective laser patterning conditions are summarized in Table 1 and Figure 3. As visually confirmed in Figure 4a, the optical transmittance of the transparent material clearly varies depending on the voids generated under different laser patterning conditions. Figure 4b further demonstrates that the void density (pitch) can be precisely controlled by adjusting the stage scanning speed.

First, examining the relationship between void pitch and transmittance shows that transmittance can be tuned from small to large variations depending on void density. This is because voids formed inside the transparent material interrupt the optical path, inducing scattering, reflection, and absorption. In particular, voids created inside transparent substrates exhibit a large refractive-index contrast relative to the surrounding medium, producing strong scattering and reflection at the void boundaries, while certain rays may undergo absorption or diffuse scattering within or near the voids [19,22]. A notable result is that transmittance decreases more sharply as the void spacing becomes denser, indicating that there exists a pitch regime in which transmittance drops rapidly. For example, decreasing the pitch from 10 μm to 7.5 μm increases the pattern density, yet the transmittance decreases by only 0.7%p at 365 nm (i-line). In contrast, when the pitch is reduced from 7.5 μm to 5 μm, or from 3 μm to 2.5 μm, the transmittance decreases by 9.2%p. and 15.8%p., respectively. Despite similar or even smaller reductions in pitch, the transmittance drops dramatically. This behavior arises because densely packed voids significantly enhance multiple scattering and interference within the medium, making light propagation more complex and increasing absorption and scattering losses. In previous studies, Yao and Nguyen [23,24] also demonstrated that in low-density conditions, scattering between individual voids is limited, so the reduction in transmitted light remains moderate. However, in high-density void configurations, rays undergo repeated scattering events, preventing it from maintaining a forward direction, causing most of it to dissipate or diffuse. From this result, it is clear that when a certain pitch is reached, a sharp drop in transmittance occurs, so more than anything else, a careful decision is needed to set the pitch for transmittance control.

#### 3.2.2. Arrangement of Pattern Layers

To achieve more effective transmittance control, the voids were arranged in two layers, and the transmittance variation associated with each layer-arrangement method was analyzed. When forming two void layers using the 20 μJ pulse energy applied previously for the pitch-effect study, scattering losses became excessively large, resulting in overly too low transmittance. Therefore, a pulse energy of 10 μJ was used for this experiment.

As shown in Figure 4c, the two layers can be arranged either in a parallel configuration or in a staggered configuration, based on the relative positions of the upper and lower void layers. In the microscope images, voids in the parallel arrangement appear darker due to the overlap of two pattern layers and are relatively sparsely distributed. In contrast, the transparent material patterned with the staggered approach exhibits alternately distinguishable dark voids (first layer) and bright voids (second layer), indicating a higher density. This difference in contrast arises from variations in focal depth when observing the upper and lower void layers through the microscope. These observations confirm that both types of void-layer arrangements were successfully implemented under experimental conditions. As summarized in Table 1, generating voids in two layers inherently reduces transmittance at a given pitch. However, the staggered configuration is more effective at reducing transmittance than the parallel configuration. Considering the in-plane propagation of light, scattering and reflection at the first void layer occur in multiple directions rather than in a single preferred direction. A portion of this scattered and reflected light continues in the forward direction and reaches the second layer. When the second-layer voids are arranged in a staggered pattern, the available transmission paths are effectively reduced to approximately pitch/2, resulting in a lower overall transmittance. The fact that transmittance decreases more prominently when the pitch is reduced from 10 μm to 5 μm further supports this interpretation in the patterning density test. Although the staggered arrangement is more effective for achieving large reductions in transmittance, precise and uniform control of the relative positions of voids in each layer is significantly difficult, as it requires highly accurate stage alignment. Therefore, from a manufacturing perspective, the parallel arrangement is more practical. For this reason, all subsequent analyses of transmittance depending on multilayer patterning and pitch were performed using the parallel configuration.

#### 3.2.3. Stack of Pattern Layers

We further examined how transmittance changes when an additional void-pattern layer is stacked in the parallel configuration at different void-pattern densities. As shown in Table 1, even with only a single additional pattern layer, the transmittance of the photomask can be effectively reduced by approximately 5–19%p. with regard to all pitch conditions. A notable observation is that the smaller the void-pattern pitch, the greater the transmittance reduction achieved by stacking an additional layer. For example, at a pitch of 5 μm, adding one more pattern layer decreases transmittance by only about 5%p., whereas at pitches of 3.5 μm and below, reductions of approximately 19%p. are obtained. This indicates that for relatively large pitch conditions, stacking multiple pattern layers is not an efficient method for reducing transmittance; instead, increasing the void density within a single layer is more effective. Additionally, for achieving large transmittance reductions, increasing the number of pattern layers under dense-pitch conditions is more effective than increasing pulse energy or further reducing pitch.

### 3.3. Critical Dimension Compensation

**Figure 5:**
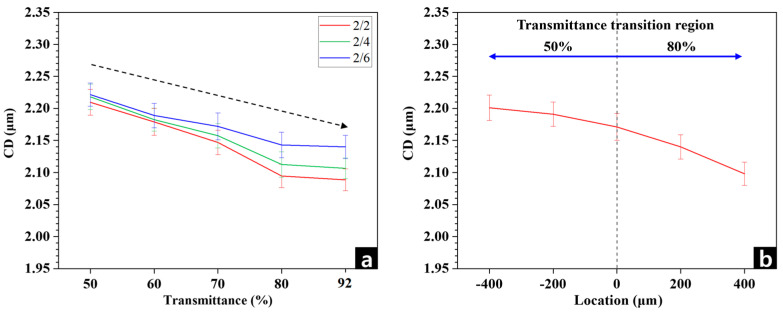
CD variation induced by linear changes in photomask transmittance and CD behavior in regions with abrupt transmittance transitions.

To analyze CD variation depending on photomask transmittances, photomasks with different transmittance levels were fabricated by forming void layers through various laser-processing conditions. In the result, reducing the transmittance of the photomask from about 92% (nonpatterned mask, bare) to 50% in steps of 10%. Figure 5 and Table 2 summarize the CD measured for different transmittance levels, space conditions, and regions exhibiting abrupt transmittance changes from 80% to 50%. Across 9 measurement regions, the overall RSD was 1.6%, and individual regions showed reasonable RSD values, confirming high reproducibility. As shown in Figure 5a and Figure S1 (Supplementary Materials), CD increases as transmittance decreases. This trend reflects the typical dose dependency (=optical intensity/time) of positive photoresists: higher transmittance increases the exposure energy delivered to the resist, enhancing dissolution in the exposed region and reducing the remaining linewidth.

The CD dependence on space shows that dense patterns (2/2) yield smaller CD than isolated patterns (2/6) under the same transmittance condition, demonstrating a characteristic iso-dense bias. This occurs because as the patterns approach each other, diffracted light interferes and increases the effective exposure energy, resulting from optical diffraction effects [25,26]. Furthermore, additional experiments on CD variation with respect to space show that, under the same transmittance conditions, relatively dense patterns (2/2) exhibit smaller CD values, demonstrating a typical iso-dense bias effect. This is because the reduced transmission efficiency of the high spatial frequency leads to increased optical loss. In the same reason, Song [27] experimentally demonstrated that when the mask open-area ratio (=transparent regions/chrome-covered regions) decreased, the line width was blurred. The author also added that rise of exposure energy is required to compensate the CDs in the dense lithographic pattern. In our data, the key point is that a transmittance changes of approximately 10% can induce control of about 30 nm level in CD. Since Figure 3 shows that laser patterning allows transmittance control on the order of a few percent, this implies that selective laser patterning can enable smooth CD correction on the scale of a few nanometers. That is, without any additional processing steps, the selective laser patterning technique applied directly inside the photomask can contribute to fine and reproducible CD compensation in the nanometer to several-tens-of-nanometers range with the slight adjustment of space.

As described in the Introduction, light-source overlap in large-area lithography affects only specific regions. Therefore, we investigated CD variation when photomask transmittance was abruptly changed in localized areas. In Figure 5b and Figure S2, the x = 0 position corresponds to the boundary between two transmittance layers, and CDs are plotted up to ±400 μm from this boundary. Contrary to the expectation that CD might rapidly deviate near a 30% transmittance discontinuity, the CD varies smoothly and gradually as far from the boundary. Moreover, farther from the boundary, CD values converge to those observed in Figure 5a, corresponding to each region’s respective transmittance level. This smooth behavior is attributed to optical smoothing: the projection system maps mask patterns onto the wafer through convolution, causing partial mixing of the 50% and 80% transmittance fields. Poonawala and Pathak [28,29] demonstrated that mask features are mapped to the wafer through an aerial-image formation process governed by spatial-frequency filtering and convolution. As a result, abrupt mask transitions are inherently smeared, since high-frequency transmittance steps are strongly attenuated by the optical transfer function. This mechanism explains why even sharp transmittance discontinuities in a photomask produce smooth and gradual CD variations on the wafer. Another reason why CD changes are smooth is that the dose–response of positive photoresist is a smooth nonlinear function governed by a dose threshold, meaning CD does not change proportionally with small dose variations. Yasin [30] reported that the resist response exhibits strong nonlinear dose-to-clear behavior, driven by acid-diffusion and threshold-based image development. Due to this nonlinear response, moderate variations in local exposure intensity do not translate proportionally into linewidth differences. Consequently, even when the photomask contains sharp transmittance contrasts, the resulting CD change on the wafer remains limited and evolves smoothly rather than abruptly. Furthermore, acid diffusion during post-exposure processes acts as a spatial smoothing factor, influencing feature shapes over hundreds of nanometers to several tens of micrometers [31]. These combined effects flatten CD variations near abrupt transmittance boundaries. Consequently, forming transmittance-control layers inside the photomask using laser patterning maintains CD stability even when transmittance gradients are present. Overall, these experimental results confirm that introducing the controllable layers of internal transmittance via laser patterning directly and effectively modifies photomask transmittance and enables smooth CD control. However, practical implementation of selective laser patterning for advanced photolithography will require detail characterizations of void morphology, theoretical modeling and simulation on scattering pattern layers, and stepwise optimization—from light-source control to precise transmittance-layer fabrication and resist-coating conditions. Future work will address these aspects to mature the technique toward production-level application dealing with large scaled photomasks.

## 4. Conclusions

In this study, we investigated a laser selective-patterning technique inside photomasks as a method to improve CD non-uniformity caused by light-source overlap in segmented large-area exposure systems. We experimentally analyzed, in detail, the morphology and limitations of voids generated under various femtosecond-laser processing conditions, the resulting changes in photomask transmittance for different void-pattern configurations, and the CD behavior after photolithography using laser-patterned photomasks. Although the void size can be controlled by adjusting the femtosecond laser pulse energy, excessive pulse energy leads to photomask damage or fracture. Photomask transmittance was found to depend strongly on void pattern density (pitch), and dense patterns can induce void overlap; under such conditions, adding additional pattern layers along the laser-beam direction enables large transmittance control. We also confirmed that CD can be precisely controlled to within a few nanometers depending on photomask transmittance and space adjustment. In regions with abrupt transmittance transitions, CD did not change abruptly but instead varied smoothly. Overall, our results demonstrate that selective femtosecond-laser patterning inside a photomask enables photomask transmittance control and smooth CD compensation. With further stepwise process optimization—from exposure-source control to precise transmittance-layer formation—the technique has strong potential for application as a CD-correction method in segmented exposure systems where light-source overlap is problematic.

## Figures and Tables

**Table 1:** Photomask transmittance depending on the pattern layers formed under various selective laser patterning conditions.

Experimental Parameters		Pulse Energy (μJ)	Layer #	Pattern Arrangement	Pitch (μm)	Transmittance (%, at 365 nm)	Application of Laser Patterning Condition for Lithography
1	Pitch	20	1	-	2.5	36.5	
					3.5	64.4	
					5.0	68.1	
					7.5	83.2	
					10.0	84.0	
2	Arrangement of pattern layers	10	2	Parallel	5	60.7	○
				Staggered	5	41.8	
				Parallel	10	79.6	○
				Staggered	10	77.4	
3	Stack of pattern layers	10	1	Parallel	2.5	50.5	○
			2			20.2	
			1		3.5	67.8	
			2			32.0	
			1		5.0	69.6	○
			2			60.7	

**Table 2:** CD measured for different transmittance levels, space conditions and locations of the transmittance transition.

Transmittance (%)	CD/Space (μm)			Location (μm)	CD (μm)
	2/2	2/4	2/6		
50	2.209	2.218	2.221	−400	2.201
60	2.179	2.182	2.189	−200	2.191
70	2.147	2.157	2.172	0	2.171
80	2.094	2.112	2.143	200	2.140
92	2.088	2.106	2.140	400	2.098

## Data Availability

Data will be made available on request.

## Supplementary Materials

The following supporting information can be downloaded at: https://www.mdpi.com/article/10.3390/mi17010095/s1, Figure S1: Photomask design schematic to evaluate CD stability and reproducibility; Figure S2: Optical microscope images of void patterns formed inside the photomask by femtosecond laser with regard to pulse energies.
