# Supplementary material for: Smooth Critical Dimension Compensation Across Photomask Transmittance Discontinuities Enabled by Selective and Direct Laser Patterning Inside Mask
Source: Micromachines (Basel). 2026 Jan 11;17(1):95. doi: 10.3390/mi17010095 (PMC12844384; doi:10.3390/mi17010095)
Supplement: Supplementary file 1 [file micromachines-17-00095-s001.zip › micromachines-4050678-supplementary.pdf]

Supplementary Materials

Smooth critical dimension compensation across photomask transmittance discontinuities enabled by selective and direct laser patterning inside mask

Dabin Park [1], Sungho Jeong[,2], Junsu Park[*,1]*

[1] School of Mechanical Engineering & Advanced Machinery Technology Research Institute, Kunsan National University, 558 Daehak-ro, Gunsan-si, Jeonbuk State, 54150, Republic of Korea

[2] Department of Mechanical and Robotics Engineering, Gwangju Institute of Science and Technology, 123 Cheomdangwagi-ro, Buk-gu, Gwangju, 61005, Republic of Korea

[*] Corresponding author

[*] Corresponding authors contributed equally to this work

[*] E-mail address: parkjs@kunsan.ac.kr (J. Park)

Journal: Micromachines

Figure S1 shows SEM images of the CDs that were quantified in Figure 5(a).

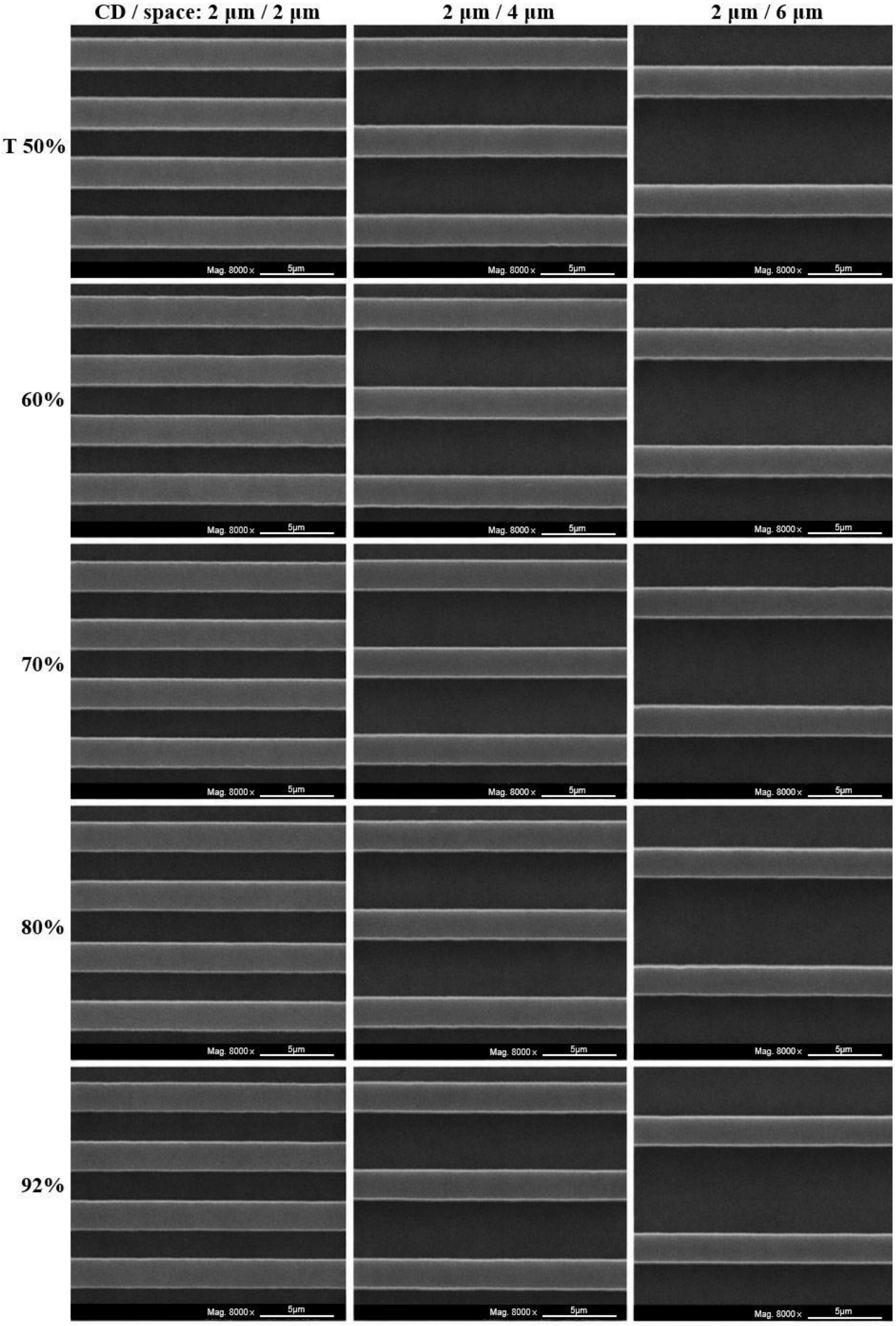

Figure S1. Photomask design schematic to evaluate CD stability and reproducibility

Figure S2 shows SEM images of the CDs in transmittance transitions that were quantified in Figure 5(b).

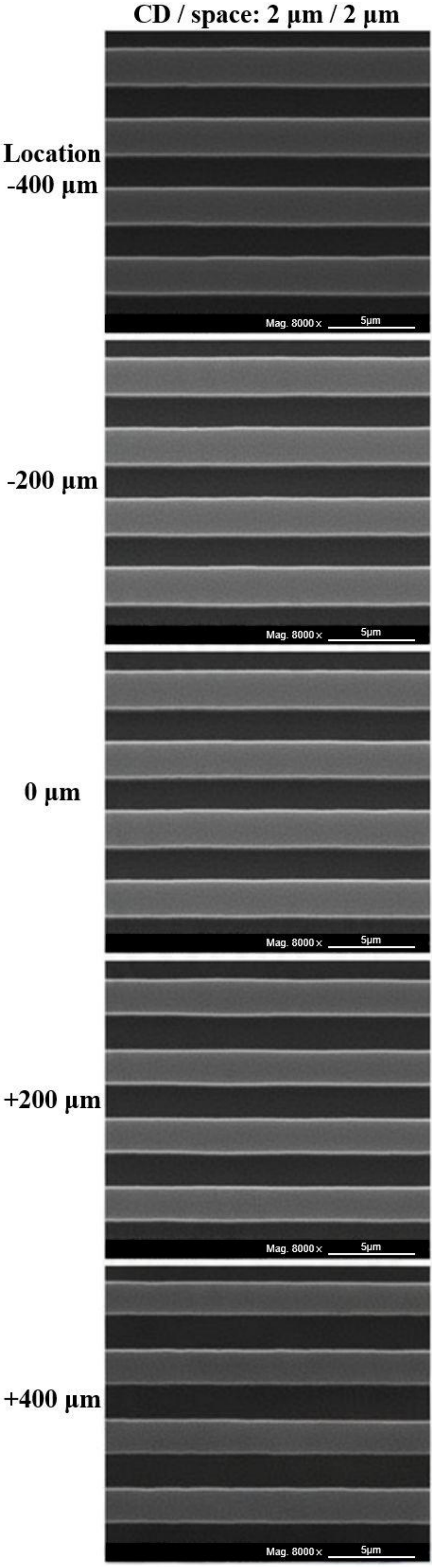

Figure S2. Optical microscope images of void patterns formed inside the photomask by femtosecond laser with regard to pulse energies
